# Searching for molecular markers in head and neck squamous cell carcinomas (HNSCC) by statistical and bioinformatic analysis of larynx-derived SAGE libraries

**DOI:** 10.1186/1755-8794-1-56

**Published:** 2008-11-11

**Authors:** Nelson JF Silveira, Leonardo Varuzza, Ariane Machado-Lima, Marcelo S Lauretto, Daniel G Pinheiro, Rodrigo V Rodrigues, Patrícia Severino, Francisco G Nobrega, Head and Neck Genome Project GENCAPO, Wilson A Silva, Carlos A de B Pereira, Eloiza H Tajara

**Affiliations:** 1 Instituto de Pesquisa e Desenvolvimento, Universidade do Vale do Paraíba, UNIVAP, São José dos Campos, SP, Brazil; 2 Instituto de Matemática e Estatística, USP, São Paulo, SP, Brazil; 3 BIOINFO-USP Núcleo de Pesquisas em Bioinformática, USP, SP, Brazil; 4 Departamento de Genética, Faculdade de Medicina de Ribeirão Preto-USP, Centro de Terapia Celular, Centro Regional de Hemoterapia, SP, Brazil; 5 Departamento de Biologia Molecular, Faculdade de Medicina de São José do Rio Preto, FAMERP, São José do Rio Preto, SP, Brazil; 6 Departamento de Genética e Biologia Evolutiva, Instituto de Biociências, USP, São Paulo, SP, Brazil; 7 Instituto de Ensino e Pesquisa Albert Einstein, São Paulo, SP, Brazil; 8 Departamento de Biociências e Diagnóstico Bucal, Faculdade de Odontologia, UNESP, São José dos Campos, SP, Brazil; 9 Complete authors list and addresses is presented in the Appendix

## Abstract

**Background:**

Head and neck squamous cell carcinoma (HNSCC) is one of the most common malignancies in humans. The average 5-year survival rate is one of the lowest among aggressive cancers, showing no significant improvement in recent years. When detected early, HNSCC has a good prognosis, but most patients present metastatic disease at the time of diagnosis, which significantly reduces survival rate. Despite extensive research, no molecular markers are currently available for diagnostic or prognostic purposes.

**Methods:**

Aiming to identify differentially-expressed genes involved in laryngeal squamous cell carcinoma (LSCC) development and progression, we generated individual Serial Analysis of Gene Expression (SAGE) libraries from a metastatic and non-metastatic larynx carcinoma, as well as from a normal larynx mucosa sample. Approximately 54,000 unique tags were sequenced in three libraries.

**Results:**

Statistical data analysis identified a subset of 1,216 differentially expressed tags between tumor and normal libraries, and 894 differentially expressed tags between metastatic and non-metastatic carcinomas. Three genes displaying differential regulation, one down-regulated (*KRT31*) and two up-regulated (*BST2*, *MFAP2*), as well as one with a non-significant differential expression pattern (*GNA15*) in our SAGE data were selected for real-time polymerase chain reaction (PCR) in a set of HNSCC samples. Consistent with our statistical analysis, quantitative PCR confirmed the upregulation of *BST2 *and *MFAP2 *and the downregulation of *KRT31 *when samples of HNSCC were compared to tumor-free surgical margins. As expected, *GNA15 *presented a non-significant differential expression pattern when tumor samples were compared to normal tissues.

**Conclusion:**

To the best of our knowledge, this is the first study reporting SAGE data in head and neck squamous cell tumors. Statistical analysis was effective in identifying differentially expressed genes reportedly involved in cancer development. The differential expression of a subset of genes was confirmed in additional larynx carcinoma samples and in carcinomas from a distinct head and neck subsite. This result suggests the existence of potential common biomarkers for prognosis and targeted-therapy development in this heterogeneous type of tumor.

## Background

Head and neck squamous cell carcinoma (HNSCC) is one of the most common malignancies in humans, affecting distinct head and neck topologies including oral cavity, oropharynx, hypopharynx, larynx and nasopharynx. HNSCC is associated with high alcohol and tobacco consumption, and represents a major international health problem with approximately 650,000 cases and 90,000 deaths per year worldwide [1]. In Brazil, over 13,000 new cases are expected in 2008 [2]. Currently, advances in both surgical and nonsurgical therapeutics have led to increased local tumor control. However, overall mortality rates have not improved due to tumor recurrences in regional and distant sites of the aerodigestive tract [3]. When detected early, HNSCC has a 75% 5-year survival rate, but most patients present metastatic disease at the time of diagnosis, which reduces survival rate to 35% [4]. This 5-year survival rate is one of the lowest among aggressive cancers and has shown no significant improvement in recent years [5,6].

Currently, there are very few molecular markers that can be used with accuracy and reliability as indicators of head and neck carcinomas with potential for metastatic progression, and therefore as indicators of a more aggressive tumor behavior. A pre-operative marker, for example, could significantly help in determining the most appropriate treatment for a particular patient [7]. Moreover, changes in the gene expression profile arising exclusively or preferentially in cancer can be used as molecular markers [8]. In fact, these markers may provide us with new means for the early detection of cancer and cancer risk assessment, as discussed by Hunter *et al*. (2005) [9] for HNSCC.

In order to investigate molecular markers that may be relevant for prognosis and therapy in cancer disease, large-scale transcriptomic approaches such as SAGE and microarrays have been extensively reported in the literature [10-12]. In the present study, we decided to use SAGE since this technique allows an unbiased global view of all the transcripts expressed in a tissue sample at a given time point. Despite its appropriateness for such studies, SAGE is an expensive and complex technique, thus commonly involving few and often rare biological samples.

We generated individual SAGE libraries from metastatic (N+) and non-metastatic (N0) larynx carcinomas, and from normal mucosa samples. A database was created to provide absolute frequency tags for each gene in metastatic and non-metastatic tumors, and for the normal tissues. For the statistical analysis of differentially expressed tags, the Poisson distribution was used as the basic probabilistic model. The Cox partial likelihood combined with Dempster p-values allowed us to consider an efficient significance test to compare the Poisson means of the three groups. Also, the choice of critical level depended on the expression power of the tag been tested. The analysis of the data by our statistical approach revealed subsets of differentially expressed genes between tumor and normal tissues, and between metastatic and non-metastatic carcinomas. These differentially expressed genes deserve further consideration as potential biomarkers for metastatic progression, and therefore as indicators of a more aggressive tumor behavior.

## Methods

### Sample preparation for SAGE and real time PCR experiments

Samples were frozen in liquid nitrogen and stored at -80°C. Total RNA was extracted using TRIzol Reagent and treated with DNase (Invitrogen Corporation, Carlsbad, CA, USA). cDNA synthesis was performed using the High Capacity cDNA Archive kit (Applied Biosystems, Forster City, CA, USA) as described by the manufacturer.

The study protocol was approved by the National Committee of Ethics in Research (CONEP 1763/05, 18/05/2005) and informed consent was obtained from all patients enrolled.

### SAGE

SAGE was carried out using the I-SAGE™ Kit (Invitrogen Corporation, Carlsbad, CA, USA). Briefly, mRNA was captured from total RNA by binding to oligo (dT) magnetic beads, and reverse transcribed with SuperScript™ II reverse transcriptase and *E. coli *DNA polymerase. Bound cDNA was cleaved with *Nla *III (anchoring enzyme), divided in two fractions and ligated to adapters A and B, both containing a *Bsm*F I restriction site followed by a CATG 3'overhang, with different primer anchoring sequences at the 5'end. Adapter linked cDNA from both fractions were cleaved with *Bsm*F I (tagging enzyme) to generate adapter linked tags that were filled in by Klenow polymerase and then mixed and ligated to form adapter linked ditags. This mixture was used as template, in three 96-well 50 μl PCR reactions using primers complementary to the adapters, and the ~100-bp products were PAGE purified. Adapters were eliminated by digestion with *Nla *III and PAGE purification of the 26 bp ditags that were ligated to form concatamers. Concatamers were submitted to polyacrylamide gel electrophoresis and regions ranging from 300–500 bp, 500–800 bp and 800–1000 bp were purified and ligated to pZero^®^-1 cloning vector. Ligation reactions were used to transform One Shot^® ^TOP10 Eletrocomp™ *E. coli *cells using 0.2 cm cuvettes and a Gene Pulser II electroporator (Bio-Rad Laboratories, Hercules, CA, USA) set at 2.5 kV, 25 mF and 200 Ω. Cells were plated on low salt LB agar containing Zeocin^®^, in plates compatible with the automated colony picker QPix2 (Genetix, New Milton, Hampshire, UK). Picked colonies were grown separately on 96-well plates containing 2XYT media. An aliquot of each well was then used directly in a PCR reaction, with forward and reverse M13 primers. Amplified inserts were checked and sequenced with forward M13 primer in a MegaBACE™1000 sequencer (Amersham Biosciences, Piscataway, NJ, USA) and the DYEnamic ET Dye Terminator Sequencing Kit (Amersham Biosciences, Piscataway, NJ, USA), or alternatively, an ABI PRISM^® ^377 DNA Sequencer (Applied Biosystems, Foster City, CA) and the ABI PRISM^® ^BigDye™ Primer Cycle Sequencing Kit (Applied Biosystems, Foster City, CA, USA).

Three SAGE libraries were generated using two larynx cancer samples (one with lymph node metastasis or N+ and one with no lymph node metastasis or N0) and a normal control library pooled from two normal samples (surgical margins from one N+ and one N0 larynx cancer). For each library, 6,000 sequencing reactions were performed and tags were extracted to yield approximately 100,000 tags per library.

The raw data files are available at the Gene Expression Omnibus database (GEO) under the accession numbers: GSM303325 (pool of normal samples); GSM303340 (N0 tumor), GSM303349 (N+ tumor).

### SAGE database

**Figure 1:**
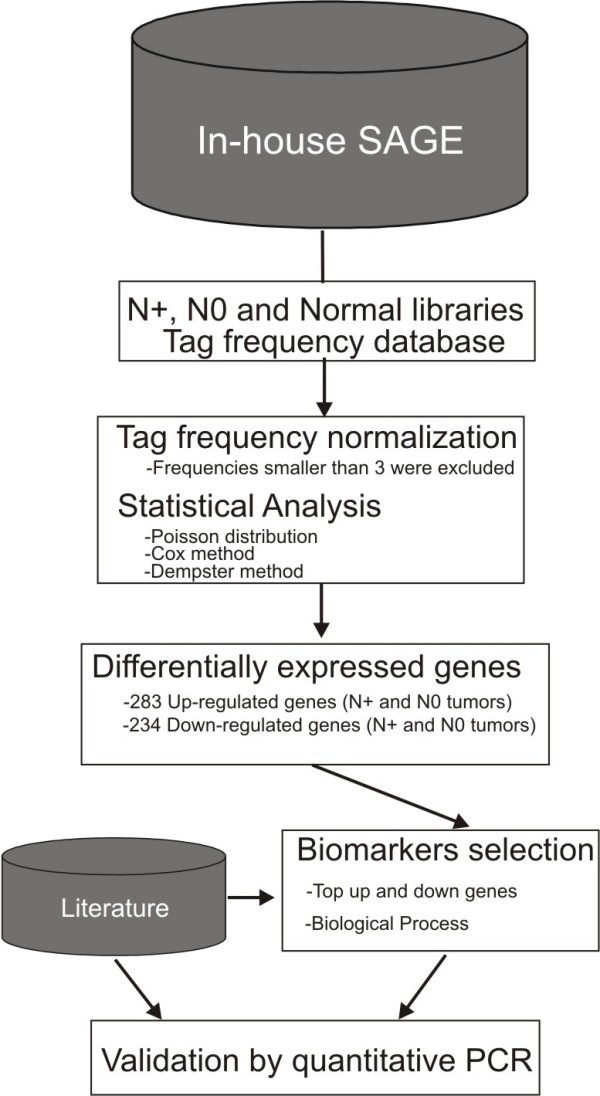
Fluxogram showing the strategy used in SAGE data analysis.

Tag frequency tables, composed of a "tag" column (10 bp sequences) and a "count" column (number of times that the tag appears in the library) were obtained by the SAGE™ Analysis 2000 Software 4.0, with minimum tag count set to 1 and maximum di-tag length set to 28 bp, whereas other parameters were set on default. A relational MySQL database [13] was developed to store data from SAGE experiments. The datasets contained information on: gene name, accession number, UniGene code, gene symbol, absolute frequency tags in metastatic and non-metastatic tumors and normal tissues. Other tables were generated to store information on metabolic pathways and gene ontology. Scripts developed in Perl [14] integrated with the MySQL database allowed the identification of genes and their respective frequencies in the three libraries which were used as input data in the program that performed statistical analysis. A schematic representation of databases, data analysis, and experimental validation representing our approach is shown in Figure 1.

### Statistical Method

Before starting the statistical analysis, we decided to exclude very low expression tags from the study. The inclusion criterion considered only tags with total normalized frequencies larger than 3. In order to obtain normalized frequencies, the absolute frequencies in each library were divided by the total number of tags of this library and multiplied by the total number of tags of the smallest library. For each tag, we observed the sum of its three normalized frequencies. If this sum was larger than 3, it was kept in the study; otherwise, it was excluded. The remaining tags, after this exclusion procedure, are the object of our study (Additional file 1, Supplementary Table 1).

Returning to the absolute frequencies of the remaining tags, we observed that all frequencies were low in relation to the size of the libraries. In such rare cases, the Poisson distribution is the adequate statistical model for the analysis. In fact, the three absolute frequencies for each tag are considered independent Poisson distributed variables. The statistical objective at this point, for a specific tag, is to decide whether there are differences in expression among the libraries. We should perform statistical tests for every tag in the data bank.

Comparing Poisson distribution is not an easy task. We then used the Partial Likelihood method as developed by Cox (1975) [15]. Briefly, the procedure considers the three frequencies of a specific tag as forming an observation of a trinomial distribution, where the sample size is now the total tag abundance, **S**. Representing now the unknown trinomial probabilities of a specific tag by (***p***_1_, ***p***_2_, ***p***_3_) and the total library sizes by (***N***_1_, ***N***_2_, ***N***_3_), homogeneity among the original three Poisson averages can be tested by testing, in the trinomial model, the hypothesis

**H: **(***p***_1_, ***p***_2_, ***p***_3_) = (***N***_1_, ***N***_2_, ***N***_3_)**/N **where **N **= ***N***_1_***+N***_2_***+N***_3_.

Again, we have a difficult task to compute a ***p-value ***in a tri-dimensional sample space. Since we have distinct tag abundances, which can go from 4 to more than one thousand tags, we have to be very precise in defining the ***p-values***. For this task, we decided to use the method developed by Dempster (1997) [16]. The method consists of ordering the sample space by the likelihood ratios. To compute the ***p-value***, the tail area was considered as the set of all points that have likelihood ratios smaller than those of the observed frequencies.

Finally, as mentioned before, the tag abundances can be very different, and considering the same significance level would be inappropriate for the tags with low frequencies. Following the recommendations of DeGroot (1975) [17], we used the decision theory optimum procedure that minimizes the risk function **aα **+ **bβ. **Here, **α **and **β **are the first and second kind of errors. In our case, we decided to choose **a = 4 **and **b = 1 **since we believe that the first type of error (deciding in favor of differentially expressed when it is not) is more dangerous than the second type of error (deciding against differentially expressed when it is). Using simulated samples, we found that the level of significance is a function of **S**, the tag abundance: **α **= **0.07S**^-1/2^.

A detailed description of the statistical method is presented in Varuzza and Pereira (2008) [18].

### Functional classification of differentially expressed genes and online gene expression analysis

Gene ontology (GO) annotation was used for the functional classification of up- and down-regulated genes. This task was performed using terms from the Gene Ontology database [19].

Additionally, we used the Oncomine database [20] in order to search for a previous association of differentially expressed genes found in this study with head and neck cancer.

### Real Time PCR

Three genes displaying down (*KRT31*) or upregulation (*BST2*, *MFAP2*) were selected for validation in additional tissues using real-time polymerase chain reaction. One gene (*GNA15*) that did not present differential expression was also selected for this validation. Their expression was checked in 26 larynx SCC samples (15 N0 and 11 N+) relative to matched normal samples and in 36 tongue SCC samples (18 N0 and 18 N+). The primers were manually designed using the following parameters: 19–23 bp length, 30–70% GC content, a short amplicon size (66–110 bp), and at least one primer of each pair flanking an intron-exon boundary to prevent genomic amplification (Table 1). All primers were purchased from Invitrogen (Brazil).

**Table 1:** Primers for real time PCR, amplicon size, values of slope, PCR efficiency and linearity (R2).

**Genes**	**Primers**	**5' → 3'**	**Amplicon size (bp)**	**Slope**	**PCR Efficiency**	**R2**
***GNA15***	Forward	GAGAACCGCATGAAGGAGAG	84	-3.312	100.0	0.991
					
	Reverse	AAAGAGGATGACGGATGTGC				

***KRT31***	Forward	TGAGCAGGAGGTCAATACCC	110	-2.913	120.4	0.990
					
	Reverse	GACTCCTGGTCTCGTTCAGC				

***BST2***	Forward	GGAGGAGCTTGAGGGAGAG	75	-3.476	93.9	0.991
					
	Reverse	CTCAGTCGCTCCACCTCTG				

***MFAP2***	Forward	GCCGTGAGGAACAGTACCC	91	-3.152	107.6	0.990
					
	Reverse	CGGAGGCTGTAGAAGCAGAC				

***TUBA6***	Forward	TCAACACCTTCTTCAGTGAAACG	101	-3.341	99.2	0.991
					
	Reverse	AGTGCCAGTGCGAACTTCATC				

***GAPDH***	Forward	ACCCACTCCTCCACCTTTGA	101	-3.392	97.1	0.990
					
	Reverse	CTGTTGCTGTAGCCAAATTCGT				

***ACTB***	Forward	GGCACCCAGCACAATGAAG	66	-3.255	102.8	0.994
					
	Reverse	CCGATCCACACGGAGTACTTG				

Real time PCR was carried out in a *7500 Real-Time PCR System *(Applied Biosystems). Reactions were performed in 20 μl with 10 μl of *Power SYBR^® ^Green PCR Master Mix *(Applied Biosystems), and 400 nM of each primer. For every experiment, 10 ng cDNA were used, and each sample was tested in triplicate. The PCR conditions were 50°C for 2 min, 95°C for 10 min followed by 40 cycles at 95° for 15 sec, 60°C for 1 min, 65°C for 34 sec. Following the PCR, dissociation curve analysis was performed to confirm the desired single gene product.

For each primer set, the efficiency of the PCR reaction (linear equation: y = slope + intercept) was measured in triplicate on serial dilutions of the same cDNA sample (a pool of 10 samples).

The PCR efficiency (E) was calculated by the formula *E *= [10^(-1/slope)^] and ranged from 1.96 to 2.02 in the different assays. The slope and *R2 *values for target and reference genes are shown in Table 1.

Initially, five control genes were used (*TUBA6*, *ACTB*, *GAPDH*, *BCR*, *HPRT*). The GeNorm program [21] calculated stability and assumed that three genes (*TUBA6*, *ACTB*, *GAPDH*) were the most appropriate.

The relative expression ratio (fold change) of the target genes was calculated according to Pfaffl (2001) [22]. Statistical analysis was calculated by a two-tailed unpaired *t *test using GraphPad prism software.

## Results and discussion

### Statistical analysis of SAGE data

We constructed three SAGE libraries from two larynx carcinoma samples and a pooled control sample aiming to identify global events involved in tumorigenesis and potential biomarkers in HNSCC.

Given the huge amount of data generated by SAGE, events that play a consistent role in cancer phenotype may be undistinguished from those that are random events, leading to false positive and false negative results. Statistical analysis and bioinformatic tools are used to overcome these limitations and improve the identification of a gene expression signature of biological and therapeutic interest. In the present study, we propose a statistical approach to analyze SAGE data through the use of Poisson probabilistic model and the conditional test of Cox partial likelihood. A Dempster methodology for ordering the sample points of the sample spaces throughout the likelihood ratio was also considered to compute the p-values. As the sample size differs considerably, we obtain the significance critical level as a function of the tag abundance. To order the differentially expressed genes we consider the relative distance of the p-values against the critical level.

A total of 53,898 SAGE unique tags were obtained: 8,979 were only found in the metastatic larynx carcinoma library, 17,588 only in the non-metastatic carcinoma library, 15,102 only in the control library, and 12,229 tags were expressed in at least two libraries (Additional file 1, Tables 2, 3, 4–5). The sequences were stored in a MySQL relational database and analyzed as shown in Figure 1. Statistical analysis identified subsets of 1,216 differentially expressed tags between tumor and normal libraries, and 894 differentially expressed tags between metastatic and non-metastatic carcinomas. Sixty top-up and 60 top-downregulated tags in aggressive versus non-aggressive tumors and in both these tumors versus normal tissues, as well as their normalized frequencies, and the corresponding genes according to SAGE Genie and SAGEmap databases [23,24] are presented in Supplementary Tables 6–11 (Additional file 2).

**Table 2:** Information on biological processes based on Gene ontology.

*Biological Process*	*Up-regulated genes*
**Cell communication**	
signal transduction	*ARHGAP29*, *BST2*, *CCL2*, *CXCL14*, *CMIP*, *FLNB*, *GNAI2*, *LY6E*
cell-cell signaling	*BST2*, *CCL2*

**Transcription**	*MZF1*, *NRG1*, *RP13-122B23.3*, *ZNF452*

**Translation**	*RPS15*, *RPS23*

**Apoptosis**	*INCA*
induction	*BID*
anti-apoptosis	*ANGPTL4*, *CCL2*, *IFI6*, *XAF1*

**Cell adhesion**	*AJAP1*, *CCL2*, *MSLN*, *SAA1*

**Cell migration**	*MYH9*, *SAA1*, *LUM*

**Cell cycle**	*PLK1*

**Cell division**	*MYH9*

**Cell proliferation**	*BOLA2*, *BST2*, *PLK1*

**Cellular development process**	
cell differentiation	*MYH9*
keratinocyte differentiation	*S100A7*, *SPRR2F*

**Cellular structure morphogenesis**	*MYH9*

**Developmental process**	*BST2*, *SPRR2F*
organ development	*CCL2*, *MEPE*, *SPARC*
epidermis development	*COL1A1*, *COL7A1*, *KRT14*, *LAMC2*, *S100A7*, *SPRR2F*
keratinization	*SPRR2F*

**Response to stimulus**	
defense response	
inflammatory response	*IL1F5*, *SERPINA3*
immune response	*BST2*, *CCL2*, *IFI6*, *IFITM2*, *IL1F5*, *SEMA3C*
response to stress	*DTL*, *SGK*
response to oxidative stress	*S100A7*
response to external stimulus	*CXCL14*, *CCL2*, *GNAI2*, *S100A7*, *SAA1*, *SEMA3C*, *TOPBP*

**Angiogenesis**	*ANGPTL4*, *MYH9*

**Transport**	*MYH9*, *NEFL*, *RBP1*, *SGK*, *SLC15A3*, *SLC6A8*

**Metabolic process**	*NADK*
protein metabolic process	*INCA*, *LEPREL1*, *MYH9*, *NRG1*, *PRSS21*, *PSMC1*
protein modification process	*CCL2*, *DTL*, *FKBP9L*, *HSPE1*, *ISG15*, *SGK*, *TOR3A*
lipid metabolic process	*APOC1*, *APOL1*, *CEL*, *PLA2G4E*, *PTGS1*, *SERPINA3*
carbohydrate metabolic process	*NANS*
DNA metabolic process	*DTL*, *H3F3B*
nucleic acid metabolic process	*0ERH*
RNA processing	*LSM4*, *SNRPD3*

**Cytoskeleton organization**	*FLNB*, *MYH9*, *NEFL*, *PLEK2*

**Extracellular structure organization**	*LUM*

**Viral genome replication**	*CCL2*

**Cellular homeostasis**	*CCL2*, *IFI6*, *SAA1*, *SELT*

**No classification**	*BASP1*, *CCNYL1*, *F8A1*, *FGFBP2*, *GRAMD1B*, *IFI27*, *KIAA1467*, *KIAA1799*, *KRTDAP*, *MFAP2*, *MSMB*, *NOL6*, *OLFML2A*, *SNCG*

	

	*Down-regulated genes*

**Cell communication**	
signal transduction	*ANXA1*, *ARHGAP27*, *CD24*, *CRABP2*, *DBNL*, *ECM1*, *GPR126*, *IL6R*, *MAL*, *TNFSF10*, *TSPAN6*, *TYRO3*
cell-cell signaling	*CD24*, *MAL*, *S100A9*, *TNFSF10*

**Transcription**	*CRABP2*, *EHF*, *HOP*, *PTRF*

**Apoptosis**	
induction	*CLU*, *MAL*, *TNFSF10*
anti-apoptosis	*ANXA1*, *SERPINB2*

**Cell adhesion**	*CLDN4*, *TYRO3*

**Cell migration**	*ANXA1*, *CD24*, *PRSS3*

**Cell proliferation**	*IL6R*, *EHF*
positive regulation	*CLU*, *TSPAN31*, *CD24*

**Cellular development process**	
cell differentiation	*CLU*, *HOP*, *KRT19*, *MAL*
keratinocyte differentiation	*A2ML1*, *ANXA1*, *SPRR3*, *TGM3*
epithelial cell differentiation	*EHF*

**Developmental process**	*EHF*, *IL6R*, *MAL*
organ development	*CLU*, *HOP*, *MAL*
ectoderm development	*KRT6A*
epidermis development	*CRABP2*, *KRT13*, *SPRR3*, *TGM3*
epidermal cell differentiation	
keratinization	*CNFN*, *PPL*, *SPRR3*

**Response to stimulus**	
defense response	*NCF1*
inflammatory response	*ANXA1*, *LYZ*, *MGLL*, *S100A8*, *S100A9*
immune response	*CLU*, *CR1*, *GBP6*, *IL1RN*, *IL6R*
response to stress	*CD24*, *CLU*
response to external stimulus	*CAT*, *CD24*, *CSTB*, *KRT8*, *PDE6B*, *SPRR3*

**Transport**	*ALDH3A1*, *AQP5*, *ARHGAP27*, *CAT*, *CD24*, *KIF1C*, *PGD*, *PLLP*, *RHCG*, *SPNS2*

**Metabolic process**	*ALDH3A1*, *CD24*, *ECHDC3*, *TPI1*
protein metabolic process	*PRSS3*, *RANBP9*, *TMPRSS11E*, *UBR4*, *USP10*
protein modification process	*ANXA1*, *PRSS3*, *TGM3*, *USP10*
lipid metabolic process	*AKR1C2*, *ANXA1*, *APOD*, *CLU*, *LTB4DH*, *MGLL*, *PIGF*, *TPI1*
carbohydrate metabolic process	*PGD*, *TPI1*

**Lymphocyte activation**	*CD24*

**Homeostasis**	*RHCG*, *CD24*

**No classification**	*C20orf149*, *C6orf205*, *C9orf58*, *CAPNS2*, *CRCT1*, *DIS3L2*, *FAM129B*, *GPRASP2*, *HPCAL1*, *IER2*, *IGHA1*, *KRT78*, *LOC342897*, *LOC643008*, *LYPD2*, *LYPD3*, *MGC59937*, *MUC1*, *NUCKS1*, *PRH1*, *TMEM59*, *TPPP3*, *ZFAND1*

Top up- and down-regulated genes selected from SAGE in tumor samples compared to normal samples.

**Table 3:** Information on biological processes based on Gene ontology.

*Biological Process*	*Up-regulated genes*
**Cell communication**	
signal transduction	*CXCL14*, *OR4S2*, *RPS6KA1*, *TNFRSF18*, *TNFSF10*
cell-cell signaling	*TOLLIP*

**Transcription**	*NRG1*, *SUMO1*

**Apoptosis**	*INCA*
induction	*TNFSF10*
anti-apoptosis	*PRKCZ*, *TNFRSF18*

**Cell-adhesion**	*MSLN*

**Cell cycle**	*CCND1*, *UBE2C*

**Cell proliferation**	
negative regulation	*EMP3*

**Cellular development process**	
cell differentiation	*KRT19*

**Developmental process**	
organ development	*KRT19*
epidermis development	

**Response to stimulus**	
defense response	
inflammatory response	*SERPINA3*
response to stress	
response to oxidative stress	*GPX2*
response to external stimulus	*CXCL14*, *OR4S2*

**Transport**	*HBB*

**Metabolic process**	*NADK*
protein metabolic process	*DKFZP586H2123*, *INCA*, *NRG1*, *SULF2*, *UBE2C*, *USP9X*
protein modification process	*CCND1*, *POMT2*, *PRKCZ*, *SUMO1*, *USP14*
lipid metabolic process	*SERPINA3*
carbohydrate metabolic process	*DCXR*
RNA metabolic process	*PCBP2*
RNA processing	*RBM17*

**DNA repair**	*SUMO1*

**No classification:**	*ANXA7*, *BRD9*, *C6orf148*, *CMIP*, *FLJ23577*, *LOC283516*, *LOC283731*, *LOC388796*, *MFAP2*, *RRP15*, *SNCG*, *TMEM109*, *ZC3H7B*

	

	*Down-regulated genes*

**Apoptosis**	*KLK8*
induction	*IGFBP3*
anti-apoptosis	*ANGPTL4*

**Cell adhesion**	*SAA1*

**Cell migration**	*SAA1*

**Cell cycle**	
Negative regulation	*KLK10*

**Cell proliferation**	
Negative regulation	*FGFBP1*
keratynocyte proliferation	*KLK8*

**Cellular development process**	
cell differentiation	*IGFBP3*, *KLK8*, *SPON2*
keratinocyte differentiation	*SPRR2E*, *SPRR2F*, *SPRR3*

**Developmental process**	*SPRR2E*, *SPRR2F*
organ development	
ectoderm development	*KRT6A*
epidermis development	*SPRR2E*, *SPRR2F*, *SPRR3*
keratinization	*SPRR2E*, *SPRR2F*, *SPRR3*

**Response to stimulus**	
defense response	*NCF1*
inflammatory response	*PI3*, *S100A8*, *S100A9,*
immune response	*DEFB4*, *HLA-A*, *PI3*, *TAPBP*
response to stress	*HIG2*, *KLK8*
response to external stimulus	*KLK8*, *SAA1*

**Angiogenesis**	*ANGPTL4*

**Transport**	*ALDH3A1*, *HBA2*, *PGD*

**Metabolic process**	*ALDH3A1*, *TPI1*
protein metabolic process	*TAPBP*
protein modification process	*CCT3*, *FKBP9L*, *HSPE1*, *IGFBP3*
lipid metabolic process	*PLA2G4E*, *TPI1*
carbohydrate metabolic process	*PGD*, *TPI1*

**Cellular homeostasis**	*SAA1*

**No classification**	*C10orf99*, *C9orf58*, *CAPNS2*, *FAM129B*, *GPRASP2*, *IGHA1*, *LMNA*, *LOC645960*, *LYPD2*, *MUC1*, *NOL6*, *PSME2*, *SLFN13*, *SNHG8*, *TJP2*, *TncRNA*

Top up- and down-regulated genes selected from SAGE in N+ tumor sample compared to N0 sample.

**Figure 2:**
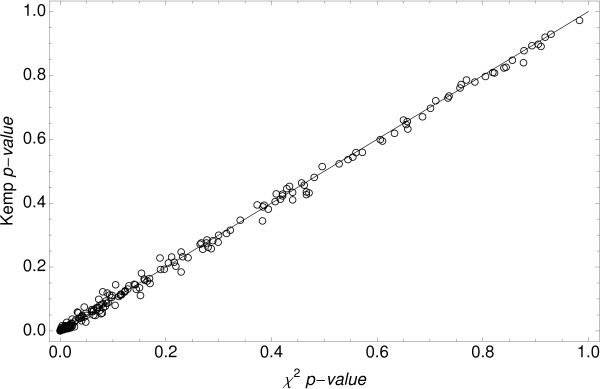
Chi-square p-value versus Kemp value for high-abundance tags.

**Figure 3:**
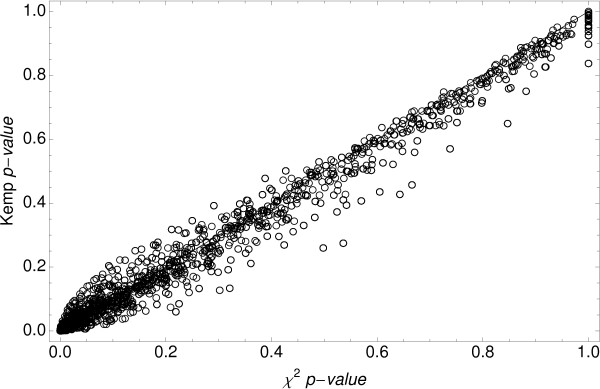
Chi-square p-value versus Kemp value for low-abundance tags.

Since several authors have reported that chi-square test is the most appropriate for SAGE experiments [25-28], we compared the performance of our statistical approach (named here as Kemp method) with that of chi-square test. For this comparison, the SAGE data set was divided into two groups: the low-abundance tags with counts equal and lower than 50, and the high abundance tags expressed at higher levels (> 50). Good correspondence between the data obtained by both tests was found for the latter tag group (Figure 2), indicating that they are equivalent for the analysis of highly expressed sequences. A similar result was not observed for low-abundance tags (Figure 3).

Using a proposed tag-customized critical level for both tests, we found 341 discordant tags, which represent 4.8% of total differentially expressed tags: 100 (29.3%) were considered differentially expressed by chi-square test but not by Kemp method, and 241 (70.7%) by Kemp but not by chi-square test. Most discordant cases were low-abundance tags (Additional file 3).

A tag presenting a differential expression pattern but low counts may be considered as statistically non-significant by methods that use fixed critical levels. Although a number of these tags probably have biological relevance, their selection from the SAGE data sets remains a challenge. To circumvent this limitation, Kemp's method calculates the critical level of each particular tag taking into account its total frequency, thus making the method applicable for detecting differences in expression of tags with counts ranging from 20 to 50. In addition, the use of a tag-customized critical level minimizes both type I and type II errors. Conversely, most of the statistical tests currently used to detect differentially expressed genes are based on asymptotic results, and perform poorly for low expression tags. Another feature of these tests is the common use of a single canonical cutoff for the significance level (p-value) of all tags, without taking into account the type II error.

### Differentially expressed genes: biological functions and potential involvement in HNSCC

Information on biological processes was obtained from the Gene Ontology (GO) database [19] for the top up- and down-regulated genes identified by the statistical approach (Tables 2 and 3). The data may be helpful for evaluating their potential as drug targets and molecular markers of cancer. Although some GO terms are not directly related to tumorigenesis, as lipid metabolism process and viral genome replication, they provide evidence of some important changes in cell metabolism coupled to energy generation and cell growth [29].

The functions of up-regulated genes in tumors include signal transduction (*BST2*, *FLNB*, *GNAI2*), transcription (*NRG1*), anti-apoptosis (*ANGPTL4*, *CCL2*, *IFI6*), cell adhesion (*SAA1*), cell migration and angiogenesis (*MYH9*), epidermis development and keratinization (*COL1A1*, *COL7A1*, *KRT14*, *LAMC2*, *S100A7*), and proteolysis (*MYH9*). Down-regulated genes are also involved in signaling (*CD24, DBNL*, *ECM1*, *TNFSF10*, *TSPAN6*), transcription (*EHF*, *PTRF*), anti-apoptosis (*SERPINB2*), keratinocyte differentiation, keratinization and epidermis development (*EHF*, *KRT13*, *SPRR3*, *TGM3*, *CRABP2*), and inflammatory response (*ANXA1*, *S100A9*). Comparison of aggressive (N+) and non-aggressive (N0) larynx tumors also showed interesting differences, including up-regulation of *NRG1*, a ligand for the receptor tyrosine kinase ErbB3 and 4 [30,31], and down-regulation of *IGFBP3 *and keratin 6A (*KRT6A*) in N+ tumor. The latter result is interesting since K6-null mice exhibit changes in the oral mucosa resembling those of congenital pachyonychia [32]. In addition, K6a/K6b double-null mice also show localized disintegration of the dorsal tongue epithelium [33]. In relation to *IGFBP-3*, which has pro-apoptotic properties [34], reduced expression has already been found in tongue SCC cases, and associated with significantly shorter disease-specific and disease-free survival [35]. The authors have suggested that its down-regulation is an early event in head and neck tumorigenesis, with adverse prognostic significance in tongue cancer, and may represent a marker of aggressive disease, reinforcing the results of the present study.

### Potential molecular markers identified by SAGE: Validation by Real-Time PCR

The selection of genes for validation by real-time RT-PCR was carried out after an extensive literature analysis of gene expression studies of head and neck carcinomas [3,4,36-66]. The following criteria were used for gene selection: (i) potential involvement in cancer development or aggressiveness and a yet unclear role in HNSCC tumorigenesis, and (ii) similar expression pattern in data reported in the literature as well as in our SAGE experiments.

Using these criteria as guidelines, four genes were selected: two with a pronounced overexpression in SAGE tumor libraries (*BST2 *and *MFAP2*), one with an intermediate downregulation profile (*KRT31*, also referred to as *KRTHA1*) and one with a non-significant differential expression pattern (*GNA15*).

According to the statistical analysis performed, *BST2 *and *MFAP2 *tags were expressed at high levels in tumors compared to normal tissues (at least 13-fold or higher), the latter also exhibiting a remarkable overexpression in N+ samples in relation to N0 samples. The normalized frequencies of *BST2 *tags showed N+ tumor/normal and N0 tumor/normal ratios of 15.8 and 24.3, respectively. For *MFAP2*, N+ tumor/N0 tumor and N+ tumor/normal ratios were 25.3 and 13.5, respectively. In contrast to these genes, *GNA15 *showed no differences in gene expression between samples analyzed by SAGE and was selected as a negative control. Although classified as a relevant underexpressed candidate marker in tumors by the statistical analysis of SAGE data, *KRT31 *displayed less expressive differences between N+ or N0 tumors and normal tissues. The normalized frequencies of tags are shown in Supplementary Tables 6–11 (Additional file 2). Similar expression patterns of *BST2*, *MFAP2*, *KRT31 *and *GNA15 *tags were observed by using a chi-square test.

**Figure 4:**
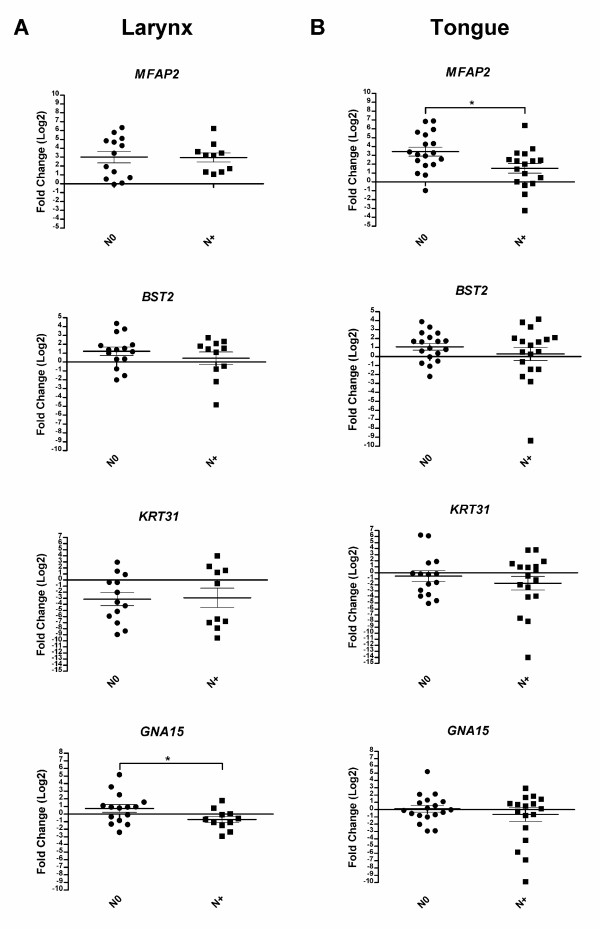
**Gene expression in metastatic (N+) and non metatastic (N0) tumors**. Expression of BST2, MFAP2, KRT31 and GNA15 was determined by real-time PCR in (A) 26 pairs of larynx tumors and matched normal tissues (15 N0 and 11 N+) and (B) 36 pairs of tongue tumor and matched normal samples (18 N+ and 18 N0). Relative quantitation of target gene expression for each sample was calculated according to Pfaffl [22]; GAPDH was used as the internal reference and normal sample as the calibrator. Values were Log2 transformed (y-axis) so that all values below -1 indicate down-regulation in gene expression while values above 1 represent up-regulation in tumor samples compared to normal samples. Differences in gene expression between groups (N0 and N+) were calculated by unpaired *t *test using GraphPad prism software and were considered statistically significant at P < 0.05 (*). The error bar represents the mean ± S.E.M (standard error of the mean).

The expression data for the selected genes were validated in 15 pairs of tumor and matched normal tissues from N0 LSCC and 11 pairs from N+ LSCC. The data were also validated in another head and neck subsite by using 36 pairs of tumor and matched normal tissues from tongue squamous cell carcinomas (18 N+ and 18 N0). *MFAP2 *was upregulated (≥ 2 fold) and *KRT31 *was downregulated (≥ 2-fold) in both N+ and N0 laryngeal tumors versus normal samples, the former also in tongue tumors. *BST2 *gene was also upregulated but only in N0 tumors versus normal tissues. No difference between N+ and N0 carcinomas was detected for these genes, except for *MFAP2 *in tongue samples. According to SAGE expression profiles, *GNA15 *exhibited a non-significant differential expression pattern in carcinomas versus normal tissues, except between N+ and N0 tumors (Figure 4).

The results of the real time PCR experiments were, therefore, in agreement with SAGE data. However, as PCR experiments were performed using a larger number of cases than SAGE, we observed high variability of gene expression among the samples. This finding suggests molecular heterogeneity in HNSCC, as previously stated by Mendez et al. (2002) [55].

The selected genes present intriguing functions related to normal and neoplastic development. *KRT31 *gene, for example, encodes a type I hair keratin, which is specifically expressed in hair and nails but has been previously observed in normal keratinocytes from buccal mucosa [67]. In cancer, loss of differentiation-specific hair keratins was found in late-stage pilomatrixoma, a skin tumor of follicular origin [68]. Since, keratin 31 has been detected in normal oral mucosa, a similar change in its expression pattern may occur in mucosa-derived squamous carcinomas.

The *BST2 *gene encodes the bone marrow stromal cell antigen 2, a transmembrane glycoprotein potentially involved in interactions between cancer cells and bone marrow stromal cells and related to angiogenesis, cell proliferation and chromosomal instability [69]. The *BST2 *promoter region contains putative *cis *elements for GATA1, STAT 3 and 1 transcription factors, the latter overexpressed in HNSCCs [70]. *BST2 *up-regulation has been observed in multiple myeloma, non-Down syndrome (DS) acute megakaryocytic leukemia, tamoxifen-resistant breast cancer [71-73] and, in the present study, was upregulated in HNSCC samples. Stromal cells prevent chemotherapy-induced apoptosis of leukemia cells [74]. The findings of Ge *et al. *(2006) [73] suggest that *BST2 *could potentially participate in the leukemia-cell protection from ara-C-induced cytotoxicity mediated by bone marrow stromal cells. These data and the findings of Becker *et al. *(2005) [72] on *BST2 *overexpression in tamoxifen-resistant breast cancer indicate that *BST2 *may possibly represent a new therapeutic target for leukemia as well as for other types of cancer, including HNSCC.

The *MFAP2 *or *MAGP-1 *gene encodes the microfibrillar-associated protein 2, a small molecular weight component of extracellular microfibrils, which are structural elements of elastic tissues in the lungs, skin, and vasculature. Miyamoto *et al*. (2006) [75] showed that MAGP-1 protein can bind to the Notch1 receptor, leading to a subsequent signaling cascade. In self-renewing tissues and during tumorigenesis, Notch signaling may inhibit differentiation, lineage specification at developmental branch points and induction of differentiation. For example, Notch signaling regulates binary cell fate decisions in the development of the peripheral nervous system in flies. Equipotent precursors give rise to two alternative cell fates: epidermal or neuronal, depending on whether a progenitor cell receives a strong or weak Notch signal. In the skin, Notch induces terminal differentiation of keratinocytes. Therefore, the Notch pathway may lead to different and sometimes opposing outcomes. One explanation is that Notch function is context-dependent [76]. Abnormal Notch activation has been observed in different tumors [77-80] although growth suppression has also been noticed after constitutively over-expressed active Notch1 [81]. Thus, Notch signaling can function as both an oncogene and a tumor suppressor, even within a single tumor, supporting the idea that the Notch1 pathway is cell-type specific and context-dependent [82].

It is noteworthy that all these three genes (*BST2*, *KRT31*, *MFAP2*) and 27 differentially expressed genes referred to above (*ANGPTL4*, *ANXA1*, *CCL2*, *CD24*, *COL1A1*, *COL7A1*, *CRABP2*, *DBNL*, *ECM1*, *EHF*, *FLNB*, *GNAI2*, *IFI6*, *KRT13*, *KRT14*, *LAMC2*, *MYH9*, *NRG1*, *PTRF*, *S100A7*, *S100A9*, *SAA1*, *SERPINB2*, *SPRR3*, *TGM3*, *TNFSF10*, *TSPAN6*) presented the same expression pattern in Oncomine data sets and in our analysis, except two genes which exhibited a different pattern (*NRG1*, *TNFSF10*) and five genes with no information in head and neck data sets available through Oncomine (*ANGPTL4*, *FLNB*, *DBNL*, *IGFBP3*, *PTRF*).

Overall, the results of the real time PCR experiments showed consistent patterns in HNSCC patients and were in agreement with SAGE analysis. However, little is known about changes at the protein level, and the relationship between gene expression and tumor phenotype as well as the potential value of these genes as biomarkers for HNSCC tumorigenesis should be evaluated in future studies.

## Conclusion

To the best of our knowledge, this is the first study reporting SAGE data in head and neck squamous cell tumors. The analysis of SAGE data by our statistical approach was effective in identifying differentially expressed genes reportedly involved in cancer development. In agreement with our statistical analysis, three genes (*BST2*, *MFAP2 *and *KRT31*) selected for validation experiments were differentially expressed in an independent subset of HNSCCs compared to normal tissues or in metastatic versus nonmetastatic samples. The selected genes have not been previously implicated in head and neck tumorigenesis. In addition, our data suggest a role for Notch signaling in HNSCC tumorigenesis, together with factors involved in keratinocyte differentiation, keratinization and epidermis development. The confirmation of the differential expression of this subset of genes selected from LSCC SAGE libraries in other HNSCC sites reinforce the existence of potential common biomarkers for prognosis and targeted therapy of such tumors.

## Competing interests

The authors declare that they have no competing interests.

## Authors' contributions

NJFS participated in the design of the study and analysis of the data, developed the expression database and bioinformatic tools and drafted the manuscript. LV participated in the analysis of the data, developed the expression database and statistical tools. AM-L, ML, PS carried out the analysis and interpretation of the data and drafted the manuscript, DGP carried out the SAGE database. RVR performed the real time PCR experiments and data analysis. FGN and GENCAPO team members were responsible for sample and data collection and initial on-site sample processing, sequencing SAGE libraries, provided the pathological analysis of the cases, obtained informed consent and discussed the findings. WASJ carried out the SAGE experiments and analysis and helped in the interpretation of data. CABP participated in the study design and coordination, carried out the analysis and interpretation of the data and drafted the manuscript. EHT participated in the study design and coordination, was responsible for sequencing SAGE libraries, carried out the analysis and interpretation of the data and drafted the manuscript. All authors read and approved the final manuscript.

## Appendix

The GENCAPO (Head and Neck Genome) Project authors are the following: Cury PM^7^, de Carvalho MB^8^, Dias-Neto E^3^, Figueiredo DLA^9^, Fukuyama EE^5^, Góis-Filho JF^5^, Leopoldino AM^15^, Mamede RCM^9^, Michaluart-Junior P^6^, Moreira-Filho CA^1^, Moyses RA^6^, Nóbrega FG^4^, Nóbrega MP^4^, Nunes FD^13^, Ojopi EPB^3^, Okamoto OK^14^, Serafini LN^10^, Severino P^1^, Silva AMA^8^, Silva Jr WA^11^, Silveira NJF^16^, Souza SCOM^13^, Tajara EH^2^, Wünsch-Filho V^12^, Zago MA^17^, Amar A^8^, Arap SS^6^, Araújo NSS^6^, Araújo-Filho V^6^, Barbieri RB^8^, Bandeira CM^4^, Bastos AU^8^, Braconi MA^4^, Brandão LG^6^, Brandão RM^11^, Canto AL^4^, Carmona-Raphe J^2^, Carvalho-Neto PB^8^, Casemiro AF^8^, Cerione M^5^, Cernea CR^6^, Cicco R^5^, Chagas MJ^4^, Chedid H^8^, Chiappini PBO^8^, Correia LA^8^, Costa A^12^, Costa ACW^8^, Cunha BR^2^, Curioni OA^8^, Dias THG^3^, Durazzo M^6^, Ferraz AR^6^, Figueiredo RO^12^, Fortes CS^12^, Franzi SA^8^, Frizzera APZ^7^, Gallo J^6^, Gazito D^8^, Guimarães PEM^6^, Gutierres AP^8^, Inamine R^12^, Kaneto CM^11^, Lehn CN^8^, López RVM^12^, Macarenco R^4^, Magalhães RP^6^, Martins AE^8^, Meneses C^4^, Mercante AMC^8^, Montenegro FLM^6^, Pinheiro DG^11^, Polachini GM^2^, Porsani AF^8^, Rapoport A^8^, Rodini CO^13^, Rodrigues AN^12^, Rodrigues-Lisoni FC^2^, Rodrigues RV^2^, Rossi L^8^, Santos ARD^11^, Santos M^8^, Settani F^5^, Silva FAM^15^, Silva IT^11^, Silva-Filho GB^6^, Smith RB^6^, Souza TB^8^, Stabenow E^6^, Takamori JT^8^, Tavares MR^6^, Turcano R^6^, Valentim PJ^5^, Vidotto A^2^, Volpi EM^6^, Xavier FCA^13^, Yamagushi F^5^, Cominato ML^5^, Correa PMS^4^, Mendes GS^5^, Paiva R^5^, Ramos O^6^, Silva C^6^, Silva MJ^5^, Tarlá MVC^11^.

**Affiliations**: ^1^Instituto de Ensino e Pesquisa Albert Einstein, São Paulo; ^2^Departamento de Biologia Molecular, Faculdade de Medicina de São José do Rio Preto; ^3^Departamento e Instituto de Psiquiatria, Faculdade de Medicina, Universidade de São Paulo (USP), São Paulo; ^4^Departamento de Biociências e Diagnóstico Bucal, Faculdade de Odontologia, Universidade Estadual Paulista, São José dos Campos, São Paulo, ^5^Serviço de Cirurgia de Cabeça e Pescoço, Instituto do Câncer Arnaldo Vieira de Carvalho, São Paulo; ^6^Departamento de Cirurgia de Cabeça e Pescoço, Faculdade de Medicina, USP, São Paulo; ^7^Departamento de Patologia, Faculdade de Medicina de São José do Rio Preto; ^8^Hospital Heliópolis, São Paulo; ^9^Serviço de Cirurgia de Cabeça e Pescoço, Faculdade de Medicina de Ribeirão Preto, USP; ^10^Departamento de Patologia, Faculdade de Medicina de Ribeirão Preto, USP; ^11^Departamento de Genética, Faculdade de Medicina de Ribeirão Preto, USP; ^12^Departamento de Epidemiologia, Faculdade de Saúde Pública, USP, São Paulo; ^13^Departamento de Estomatologia, Faculdade de Odontologia da USP, São Paulo; ^14^Departamento de Neurologia/Neurocirurgia, UNIFESP, São Paulo; ^15^Departamento de Análises Clínicas, Toxicológicas e Bromatológicas, Faculdade de Ciências Farmacêuticas de Ribeirão Preto, USP; ^16^Instituto de Pesquisa e Desenvolvimento, UNIVAP, São José dos Campos; ^17^Departamento de Clínica Médica, Faculdade de Medicina de Ribeirão Preto, USP, SP, Brazil.

## Pre-publication history

The pre-publication history for this paper can be accessed here:



## Supplementary Material

Additional file 1 **Supplementary Table 1.** Tags with different frequencies between larynx SAGE libraries according to the criteria described in the Materials and Methods section.
Supplementary Table 2. A total of 8,979 tags expressed in the N+ tumor SAGE library.
Supplementary Table 3. A total of 17,588 tags expressed in the N0 tumor SAGE library.
Supplementary Table 4. A total of 15,102 tags expressed in the control SAGE library.
Supplementary Table 5. A total of 12,229 tags expressed in at least two SAGE libraries. Click here for file

Additional file 2 **Supplementary Table 6.** Sixty top-up regulated tags in aggressive versus non-aggressive larynx library.
Supplementary Table 7. Sixty top-down regulated tags in aggressive versus non-aggressive larynx library.
Supplementary Table 8. Sixty top-up regulated tags in aggressive versus normal larynx library.
Supplementary Table 9. Sixty top-down regulated tags in aggressive versus normal larynx library.
Supplementary Table 10. Sixty top-up regulated tags in non-aggressive versus normal larynx library.
Supplementary Table 11. Sixty top-down regulated tags in non-aggressive versus normal larynx library. Click here for file

Additional file 3 **Supplementary Table 12.** Discrepancies between Kemp and chi-square analysis of SAGE data set. Click here for file
